# TLR4-dependent activation of dendritic cells by an HMGB1-derived peptide adjuvant

**DOI:** 10.1186/1479-5876-12-211

**Published:** 2014-08-14

**Authors:** Rebecca Saenz, Diahnn Futalan, Lien Leutenez, Fien Eekhout, Jessie F Fecteau, Simeon Sundelius, Stig Sundqvist, Marie Larsson, Tomoko Hayashi, Boris Minev, Dennis Carson, Sadik Esener, Bradley Messmer, Davorka Messmer

**Affiliations:** Rebecca and John Moores Cancer Center, University of California San Diego (UCSD), 3855 Health Sciences Dr., La Jolla, CA 92093-0815 USA; Pharmaceutical and Biological Laboratory Technology, University College Ghent, Ghent, Belgium; Molecular Virology, Department of Clinical and Experimental Medicine, Linköping University, Linköping, Sweden; Genelux Corporation, San Diego, CA USA; Department of NanoEngineering, UCSD, La Jolla, CA USA

## Abstract

High mobility group box protein 1 (HMGB1) acts as an endogenous danger molecule that is released from necrotic cells and activated macrophages. We have previously shown that peptide Hp91, whose sequence corresponds to an area within the B-Box domain of HMGB1, activates dendritic cells (DCs) and acts as an adjuvant *in vivo*. Here we investigated the underlying mechanisms of Hp91-mediated DC activation. Hp91-induced secretion of IL-6 was dependent on clathrin- and dynamin-driven endocytosis of Hp91 and mediated through a MyD88- and TLR4-dependent pathway involving p38 MAPK and NFκB. Endosomal TLR4 has been shown to activate the MyD88-independent interferon pathway. Hp91-induced activation of pIRF3 and IL-6 secretion was reduced in IFNαβR knockout DCs, suggesting an amplification loop via the IFNαβR. These findings elucidate the mechanisms by which Hp91 acts as immunostimulatory peptide and may serve as a guide for the future development of synthetic Th1-type peptide adjuvants for vaccines.

**Electronic supplementary material:**

The online version of this article (doi:10.1186/1479-5876-12-211) contains supplementary material, which is available to authorized users.

## Introduction

The adaptive immune response is commonly initiated via pathogen-associated molecular patterns (PAMPs) that are recognized by Toll-like receptors (TLRs) [1]. Dendritic cells (DC) are central for the initiation of adaptive immune responses and are activated by exogenous PAMPs such as lipopolysaccharides (LPS), CpG, or poly(I:C) [2] as well as endogenous signals of tissue and cell damage, sometimes referred to as alarmins or danger signals [3, 4]. Alarmins can take the form of inflammatory cytokines secreted by cells proximal to the site of injury [5] or internal components of damaged cells. Evidence for the latter includes reports that necrotic cell lysates, more specifically heat shock proteins (HSPs) and high mobility group box protein 1 (HMGB1) in the lysates, can induce DC maturation [6–8].

HMGB1 was originally described as a nuclear protein that facilitates DNA bending and stabilizes nucleosome formation [9]. HMGB1 contains three domains, including two homologous DNA binding motifs termed A- and B-boxes, each approximately 80 amino acids long, and a negatively charged C-terminus [10, 11]. In addition to the nuclear functions, HMGB1 is secreted by both macrophages and monocytes after exposure to LPS, TNF-α or IL-1β [12] and, through a feedback loop, acts back on monocytes by stimulating the synthesis of additional pro-inflammatory cytokines [13]. More recently, HMGB1 was identified as an endogenous alarmin, or damage-associated molecular pattern (DAMP) [3, 14]. HMGB1 is released from necrotic cells to trigger inflammation [15] and act as an endogenous adjuvant [16]. Several receptors are implicated in HMGB1 mediated activation of cells, including the receptor for advanced glycation end-products (RAGE) [17, 18], toll like receptor 2 (TLR2), TLR4 [19–23], TLR9 [24], Mac-1 [25], syndecan-1 [26, 27], receptor-type tyrosine phosphatase-ζ/β [26, 28], and CD24/Siglec-10 [29].

Structure-function studies have revealed that the pro-inflammatory domain in HMGB1 maps to the B-box domain, which recapitulates the cytokine activity of full-length HMGB1 [30, 31]. We have previously shown that a B-box domain derived peptide, named Hp91 acted as a potent maturation stimulus for DCs and induced a cytokine profile typical of a Th1-type response [32]. We recently showed that Hp91 potentiates antigen-specific humoral and cellular immune responses *in vivo* [33]. This study explored the mechanism by which Hp91 activates antigen presenting cells by investigating cellular uptake, receptor dependence, and signaling pathways. We found that Hp91-induced secretion of IL-6 was mediated through a MyD88/TLR4-dependent pathway involving p38MAPK and NFκB.

## Materials and methods

### Animals

Female C57BL/6 mice 8–12 weeks of age were used for experiments. C57BL/6 mice were purchased from Charles River Laboratories (Boston, MA, USA). TLR4-/- and IL1R-/- mice were purchased from The Jackson Laboratories (Bar Harbor, ME, USA). IFNαβR-/- mice were purchased from B&K Universal (England, UK). MyD88-/- and TLR7-/- mice were a gift from S. Akira (Osaka University, Osaka, Japan) and backcrossed for 10 generations onto the C57BL/6 background. Mice were bred and maintained at the Moores UCSD Cancer Center animal facility and all animal studies were approved by the Institutional Animal Care and Use Committee of UCSD and were performed in accordance with the institutional guidelines.

### Reagents

The peptides, including Hp91 (DPNAPKRPPSAFFLFCSE), Hp121 (SIGDVAKKLGEMWNNTAA), scrambled Hp91 (ASLAPPFPNCFDPKSREF), and OVA-I (SIINFEKL) were all synthesized at GMP facilities by GenScript Corp (Piscataway, NJ, USA) and CPC Scientific (San Jose, CA, USA). Peptides were synthesized with an N-terminal biotin, acetyl, or fluorescent tag (Cp488) as indicated in the figure legends. Peptides were routinely synthesized with greater than 95% purity. Peptides, reagents, and labware were endotoxin-free as determined by the manufacturer or a limulus amoebocyte assay (LAL) (Cambrex Corporation, East Rutherford, NJ), tested according to manufacturer’s instructions. Peptides were dissolved in RPMI or PBS for *in vitro* and *in vivo* experiments respectively. Phenylarsine oxide and chlorpromazine (clathrin-mediated endocytosis inhibitors), sodium azide (energy-dependent endocytosis inhibitor), nystatin (caveolin-mediated endocytosis inhibitor), latrunculin B (phagocytosis inhibitor), amiloride (micropinocytosis inhibitor), and dynasore (dynamin inhibitor) were purchased from Sigma-Aldrich as endocytosis inhibitors. The p38 MAPK-specific inhibitor, SB203580, and the NFκB inhibitor, *N*-tosyl-L-phenylalanine chloromethyl ketone (TPCK), were purchased from Sigma-Aldrich. The MEK1 inhibitor, PD98059, was purchased from Cell Signaling Technology (Danvers, MA). As many of these inhibitors required solubilization in DMSO, DMSO was used as a negative control.

### Cell lines

The J774 cell line was a gift from Maurizio Zanetti (UCSD) and was cultured in RPMI 1640 medium (Invitrogen), supplemented with 10 mM HEPES (Invitrogen), penicillin (100 U/ml), streptomycin (100 μg/ml), L-glutamine (2 mM) (Invitrogen), and 10% (vol/vol) fetal calf serum (Omega Scientific, Tarzana, CA). The RAW 264.7 cell line was a gift from Dong-Er Zang (UCSD) and was cultured as above, except with 5% (vol/vol) fetal calf serum (Omega).

### Generation of human monocyte-derived DCs

Peripheral blood mononuclear cells were isolated from the blood of normal volunteers over a Ficoll-Hypaque (Amersham Biosciences, Uppsala, Sweden) density gradient. Anonymous blood samples were purchased from the San Diego Blood Bank; therefore, no institutional review board approvals were necessary. To generate DCs, peripheral blood mononuclear cells were allowed to adhere to culture plates for 1 h. The non-adherent cells were washed off, and the adherent cells were cultured in RPMI 1640 medium (Invitrogen) supplemented with 50 mmol 2-mercaptoethanol (Sigma-Aldrich), 10 mM HEPES (Invitrogen), penicillin (100 U/ml), streptomycin (100 μg/ml), L-glutamine (2 mM) (Invitrogen), and either 5% (vol/vol) human AB serum (Gemini Bio Products, West Sacramento, CA) or 1% (vol/vol) human plasma (Valley Biomedical, Winchester, VA) and supplemented with GM-CSF (1000 U/ml) (Bayer HealthCare Pharmaceuticals, Wayne, New Jersey), and interleukin-4 (100 U/ml) (IL-4; R&D Systems, Minneapolis, Minnesota) at days 0, 2, and 4. Immature human DCs (iDCs) were harvested on days 5–7.

### Generation of mouse bone marrow-derived DCs

Bone marrow-derived DCs (BM-DCs) were prepared from C57BL/6 and knockout mice, as described by Inaba et al. [34] with minor modifications. Briefly, single bone marrow cell suspensions were obtained from femurs and tibias and depleted of lymphocytes, granulocytes, and Ia + cells by incubating with a mixture of monoclonal antibodies (mAbs; anti-CD4, anti-CD8, anti-B220/ CD45R, and anti-Ia) (antibody hybridomas were a gift from Ralph Steinman (Rockefeller)) and low-toxicity rabbit complement (Pel Freez Biologicals, Rogers, AR) for 60 min at 37°C. Cells were re-suspended at a concentration of 10^6^ cells/ml in RPMI 1640 medium (Invitrogen) supplemented with 50 mM 2-mercaptoethanol (Sigma-Aldrich), 10 mM HEPES (Invitrogen), penicillin (100 U/ml), streptomycin (100 μg/ml), L-glutamine (2 mM) (Invitrogen), 5% (vol/vol) fetal calf serum (Omega), and 10 ng/ml recombinant murine granulocyte-macrophage colony-stimulating factor (GM-CSF) (J558L GM-CSF-secreting cells were a gift from Ralph Steinman). Fresh complete medium containing GM-CSF was added on days 2 and 4 of culture. Cells were collected for the experiments on days 5–7.

### Confocal microscopy

1x10^5^ immature human DCs were precooled on ice and subsequently incubated for 30 min on ice with biotinylated-Hp91 or Hp121 to allow peptide binding. Cells were washed and then incubated for the indicated time at 37°C. Cells were cytospun (Shandon Cytospin 2 centrifuge) onto glass slides, fixed, permeabilized with acetone, and stained with Streptavidin-Alexa 488 (Invitrogen) to visualize biotinylated peptides and Hoechst 33258 (Invitrogen) to visualize DNA. Cells were imaged on a Zeiss LSM confocal microscope.

### Binding/uptake studies

For most experiments, iDCs or mouse BM-DCs were precooled on ice for 30 min, as indicated in the figure legends. Cells were subsequently incubated for the indicated times and temperatures in culture medium with biotinylated peptides. Cells were washed, permeabilized with Cytofix/Cytoperm (BD Biosciences, Franklin Lakes, NJ), stained with Streptavidin-Alexa 488 (Invitrogen), and analyzed by flow cytometry. For experiments with endocytosis inhibitors, cells were pre-treated for 30 min with the indicated inhibitors or controls prior to incubation with the biotinylated peptides. For experiments with J774 mouse macrophages, cells were pre-cooled on ice, pre-treated with 30 min with the indicated inhibitors, and subsequently incubated for 30 min with fluorescently-labeled Hp91 (Cp488-Hp91). Cells were immediately analyzed by flow cytometry using the FACSCalibur (Beckon Dickinson, Franklin Lakes, NJ). Data were analyzed using the FlowJo software (Tree Star, Inc., Ashland, OR).

### Stimulation of DCs

At days 5–7 of culture, DCs were either left untreated or were stimulated with indicated doses of peptide. For inhibition experiments, immature human DCs were pretreated with the indicated doses of SB203580, PD98059, *N*-tosyl-L-phenylalanine chloromethyl ketone (TPCK), or DMSO control for 30 min prior to stimulation. For experiments with human DCs, supernatants were collected 48 h after stimulation and the level of IL-6 analyzed by IL-6 ELISA (eBioscience, Inc. San Diego, CA). For experiments with mouse BM-DCs, supernatants were analyzed by ELISA (eBioscience), 24 h after stimulation.

### Immunizations and splenocyte preparation

Mice were immunized s.c. with 50 μg of OVA-derived SIINFEKL peptide (OVA-1). The SIINFEKL peptide was co-administered with PBS, Hp91 (250 μg), or scrambled Hp91 (250 μg). Peptides were re-suspended in PBS for all immunizations. Mice were boosted two weeks later and spleens and blood were collected one week after the final immunization. Single cell suspensions of splenocytes were prepared by mechanical disruption and separation through a 70 mm nylon cell strainer (BD Biosciences). Red blood cells were lysed using ammonium chloride buffer (Roche Diagnostics, Indianapolis, IN) and the splenocytes were subsequently re-suspended in RPMI 1640 medium (Invitrogen) supplemented with 10 mM HEPES (Invitrogen), penicillin (100 U/ml), streptomycin (100 μg/ml), L-glutamine (2 mM) (Invitrogen), and 5% (vol/vol) fetal calf serum (Omega).

### Enzyme-linked immunospot assay

Freshly isolated splenocytes were plated in duplicate wells in an Immobilon-P (PVDF) bottom enzyme-linked immunospot (ELISpot) plate (Millipore, Millerica, MA, USA) precoated with 5 μg/ml monoclonal anti-mouse IFN-γ antibody (Mabtech, Stockholm, Sweden). Splenocytes were cultured overnight at 37°C with 2.5 μg/ml SIINFEKL (OVA-I) peptide, 2.5 μg/ml ISQAVHAAHAEINEAGR (OVA-II) peptide, 5 μg/ml concanavalin A positive control (Sigma-Aldrich), or left unstimulated (medium only). After 18 h, culture supernatants were collected for cytokine analysis and ELISpot plates were developed using 1 μg/ml biotinylated anti-mouse IFN-γ antibody (Mabtech), Streptavidin-HRP, and TMB Substrate (Mabtech). The plate was scanned and the spots were counted using an automated ELISpot Reader System (CTL ImmunoSpot, Shaker Heights, OH, USA).

### Cytokine Release Assay

Splenocytes were cultured overnight with 2.5 μg/ml OVA-I peptide, 5 μg/ml concanavalin A positive control (Sigma-Aldrich), or left unstimulated (media only). After 18 h, cell culture supernatants were collected and analyzed for the presence of IL-2 by ELISA (eBioscience).

### Immunoblotting

Mouse J774 or RAW 264.7 macrophages stimulated with Hp91 or LPS for 20, 40, or 60 minutes were lysed for 20 minutes on ice in RIPA lysis buffer (10 mM Tris pH 7.4, 150 mM NaCl, 1% TritonX-100, 0.1% sodium deoxycholate, 0.1% sodium dodecylsulfate (SDS), 5 mM EDTA supplemented with 1 mM phenylmethylsulfonyl fluoride, Halt phosphatase inhibitor (Thermo Fisher Scientific, Rockford, IL), 1 mM sodium vanadate, 1 mM sodium fluoride, and complete protease inhibitor cocktail (Roche). Protein concentration was determined with the Detergent Compatible protein assay (Bio-Rad, Hercules, CA). The lysates were snap-frozen and stored at -80°C. Equal amounts of protein lysates were separated by gel electrophoresis with the use of a NuPAGE Novex 4%-12% Bis-Tris Midi Gel (Invitrogen) and transferred to polyvinylidene fluoride membranes (Bio-Rad). Membranes were washed with 1 × TBST (Tris-Buffered Saline Tween-20) and blocked for 1 hour at room temperature in 5% milk/TBST. Membranes were probed overnight for phospho- p38, phospho (p)-interferon regulatory factor 3 (IRF3), p38, IRF3, glyceraldehyde-3-phosphate dehydrogenase (GAPDH), or β-actin (Cell Signaling Technology). The next day, membranes were washed with 1× TBST and incubated with goat anti–rabbit or anti–mouse horseradish peroxidase–conjugated secondary antibodies (Santa Cruz Biotechnology, Santa Cruz, CA) diluted in 5% milk/TBST for 1 h at room temperature. Antibodies were detected with the use of either an enhanced chemiluminescence detection kit (GE Healthcare, Piscataway, NJ) or SuperSignal West Femto Maximum Sensitivity Substrate (Thermo Fisher Scientific). In some experiments, cells were pre-treated with the endocytosis inhibitor Dynasore.

### Qualitative real-time PCR

Qualitative real-time PCR (qPCR) was performed in a Stratagene Mx3005P (Agilent, Santa Clara, CA) for mouse IFN- α2 and GAPDH. GAPDH was used as an endogenous standard for normalization of the IFN- α2 gene. Briefly, 1.25 × 10^5^ J774 macrophages/well were serum-starved overnight in a 96-well flat-bottom plate and stimulated in duplicate with LPS (10 ng/ml), acetylated Hp91 (200 μg/ml), or left unstimulated (media) for 6 hours. Cells were harvested and RNA was isolated using TRIzol as follows: the J774 cell pellets were lysed in approximately 1 ml of TRIzol Reagent (Invitrogen) by repetitive pipetting. The cleared homogenate solution was incubated for 5 min at RT, 200 μl of chloroform was added and samples were shaken for 15 seconds and incubated at RT for an additional 2–3 min. Samples were centrifuged at 12000 × g for 15 minutes at 4°C. Pellets were washed with 1 ml 75% RNAse-free ethanol, centrifuged for 7000 × g for 5 min at 4°C, and the RNA pellets were air dried. DNase was removed from samples using a Turbo DNAfree DNase treatment (Applied Biosystems/Ambion, Austin, TX). cDNA was synthesized using Superscript III-RT polymerase (Invitrogen) and related reagents as per the manufacture’s instructions. qPCR samples were setup using Brilliant II SYBR Green QPCR Master Mix (Invitrogen) and the following Q-primers: IFN-α2 (For. 5‘-ACTCTGTGCTTTCCTCGTGATGCT-3’; Rev. 5‘-ATCCAAAGTCCTGCCTGTCCTTCA-3’) and GAPDH (For. 5‘- TCACCACCATGGAGAAGGC-3’; Rev. 5‘-GCTAACCAGTTGGTGGTGCA-3’). Primers were purchased from IDT. qPCR was performed on duplicate samples in a Stratagene Mx3005P. Amplification product lengths were confirmed on a DNA gel. Values are normalized against GAPDH controls.

### Statistical analysis

Data were analyzed for statistical significance using unpaired or paired Student’s *t*-test or the Log Rank test. Statistical analysis was performed using GraphPad software version 5.01 for Windows (GraphPad Software, San Diego, CA, USA). A *p* value <0.05 was considered statistically significant.

## Results

### Hp91 amino acid sequence is critical for uptake and activation of DCs

**Figure 1:**
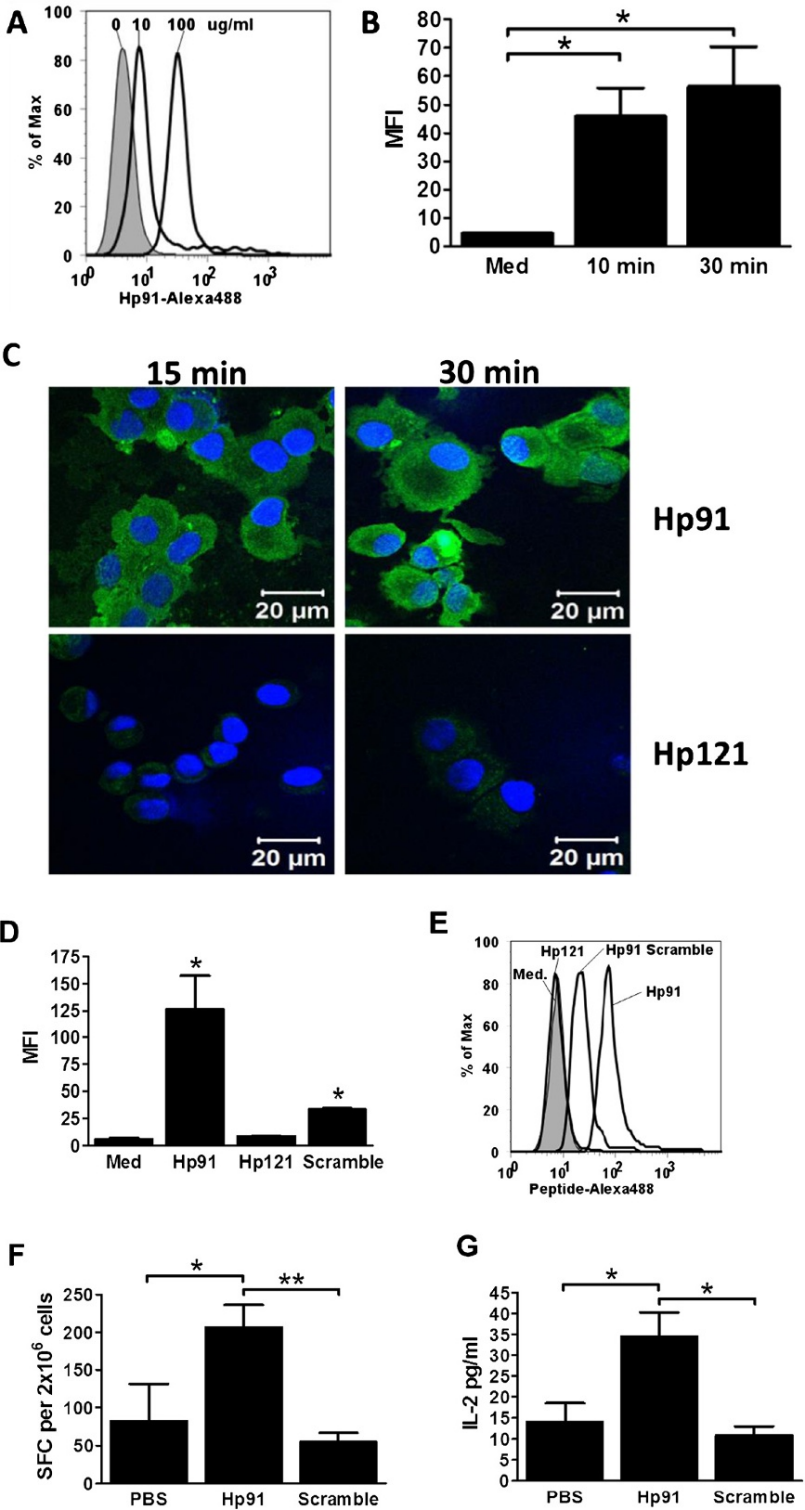
***Hp91 uptake by DCs is dose, time, and sequence dependent*** **. (A)** Immature human DCs were pre-incubated on ice for 30 min, then incubated with biotinylated-Hp91 (0, 10, or 100 μg/ml) for 30 min at 37°C. Cells were permeabilized with Cytofix/Cytoperm, stained with Streptavidin-Alexa 488, and analyzed by flow cytometry. The results shown is one representative experiment (N = 6). **(B)** Immature human DCs were incubated with 100 μg/ml biotinylated Hp91 for 10 or 30 minutes, permeabilized, stained, and analyzed as above. Results shown are mean (±SEM) of N = 4. **(C)** Pre-cooled immature human DCs (iDCs) were incubated on ice for 30 min with 200 μg/ml biotinylated Hp91 or Hp121 to allow peptide binding, washed, then incubated for 15, or 30 additional min at 37°C. Cells were cytospun, fixed, permeabilized, and stained with Streptavidin-Alexa 488 to visualize biotinylated peptides (Green) and Hoechst DNA stain (Blue). Cells were imaged on a Zeiss LSM confocal microscope. Data shown is one representative experiment of N = 3. **(D-E)** Immature human DCs were pre-cooled on ice for 30 min, then incubated with media only, biotinylated-Hp91, biotinylated-Hp121, or biotinylated-scrambled Hp91 (“Scramble”) at 200 μg/ml for 30 min at 37°C. Cells were permeabilized, stained, and analyzed by flow cytometry as above. **(D)** is mean (±SEM) for N = 3 and **(E)** is one representative experiment. *p < 0.05 compared to medium; Student’s t-test. **(F, G)** Mice were immunized with OVA-I peptide in PBS with Hp91 or scrambled Hp91 peptide (250 μg). **(F)** Freshly isolated splenocytes from the immunized mice were examined in an OVA IFN-γ ELISpot assay. **(G)** Supernatants were collected and analyzed for IL-2 secretion by ELISA. The data shown is mean (±SEM) for 5–10 mice/group. *p < 0.05 between groups; Student’s t-test.

To gain insight into the mechanism of action of the DC stimulatory peptide Hp91, we investigated its physical interaction with DCs. Hp91 was taken up in a dose dependent manner (Figure 1A), which plateaued between 10 and 30 minutes (Figure 1B). The control peptide, Hp121, which also corresponds to a sequence present in HMGB1 and has the same length, a similar charge, and isoelectric point as Hp91 was not taken up by DCs and uptake of a scrambled version of Hp91, was significantly lowered (Figure 1C, 1D and 1E), suggesting that uptake of Hp91 is sequence specific and not due to overall charge. Confocal microscopy was used to distinguish between cellular binding and uptake and showed localization of Hp91 inside DCs. The control peptide Hp121 was not detected inside the cells, even after 30 minutes incubation at 37°C (Figure 1C). Since the scrambled version of Hp91 interacted with DCs, although to a much lesser extent than Hp91 (Figure 1D and 1E), we examined if it had similar immunogenic properties as Hp91, which as we have previously described potentiates antigen-specific CD8+ T cell immune responses *in vivo* [33]. The scrambled version of Hp91 did not augment OVA-specific CD8+ CTL responses or IL-2 release (Figure 1F and 1G), suggesting that Hp91 functional activity is sequence specific.

### Hp91 enters dendritic cells via clathrin-mediated endocytosis

**Figure 2:**
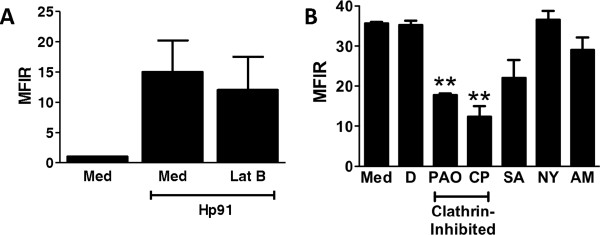
**Uptake of Hp91 is via clathrin-mediated endocytosis. (A)** Immature human DCs were pre-treated with the phagocytosis inhibitor Latrunculin B or medium only for 30 min before incubation with biotinylated-Hp91 for 30 min. Cells were permeabilized with Cytofix/Cytoperm, stained with Streptavidin-Alexa 488, and analyzed by flow cytometry. Data are mean (±SEM) of N = 5. **(B)** Immature human DCs were pre-treated for 30 min with media only (Med), DMSO (D) = solvent control, the clathrin-mediated endocytosis inhibitors phenylarsine oxide (PAO) at 2 μM or chlorpromazine (CP) at 100 μM, the energy-dependent endocytosis inhibitor sodium azide (SA) at 10 mM, the caveolin-mediated endocytosis inhibitor nystatin (NY) at 20 μM, or the micropinocytosis inhibitor amiloride (AM) at 2 mM before incubation with biotinylated Hp91 (200 μg/ml) for 30 min. Cells were permeabilized, stained, and analyzed by flow cytometry as above. Results are mean (±SEM) of N = 3. **p < 0.01 compared to DMSO control; Student’s t-test.

Hp91 was taken up at 37°C, but not at 16 and 4°C (Additional file 1: Figure S1), indicating that the uptake occurred via an energy dependent process, i.e. endocytosis. This was further supported by a significant inhibition of uptake of a fluorescently labeled version of Hp91 (Cp488) by sodium azide, which inhibits energy-dependent endocytosis (data not shown). The endocytic pathways include phagocytosis, macropinocytosis, clathrin-mediated endocytosis, and lipid-raft/caveolin-mediated endocytosis. The mechanism involved in the Hp91 uptake was determined using specific inhibitors of these endocytosis pathways. Latrunculin B (LatB), a specific phagocytosis inhibitor, did not reduce uptake of Hp91 (Figure 2A), whereas it completely abrogated uptake of the control Dextran FITC (data not shown). Phenylarsine oxide and chlorpromazine, inhibitors of clathrin-mediated endocytosis, significantly reduced the uptake of Hp91, suggesting that uptake occurs via clathrin-mediated endocytosis (Figure 2B). In contrast, no effects were seen on the uptake of Hp91 for the lipid-raft/caveolin-mediated endocytosis inhibitor, nystatin, or the macropinocytosis inhibitor, amiloride (Figure 2B), indicating that the uptake was not occurring via neither lipid rafts nor macropinocytosis. These data show that the major mechanism for Hp91 uptake into DCs occurs via a clathrin coated pit dependent manner.

### Hp91-mediated activation of DCs *in vitro*is dependent on TLR4, MyD88, and IFNαβR

**Figure 3:**
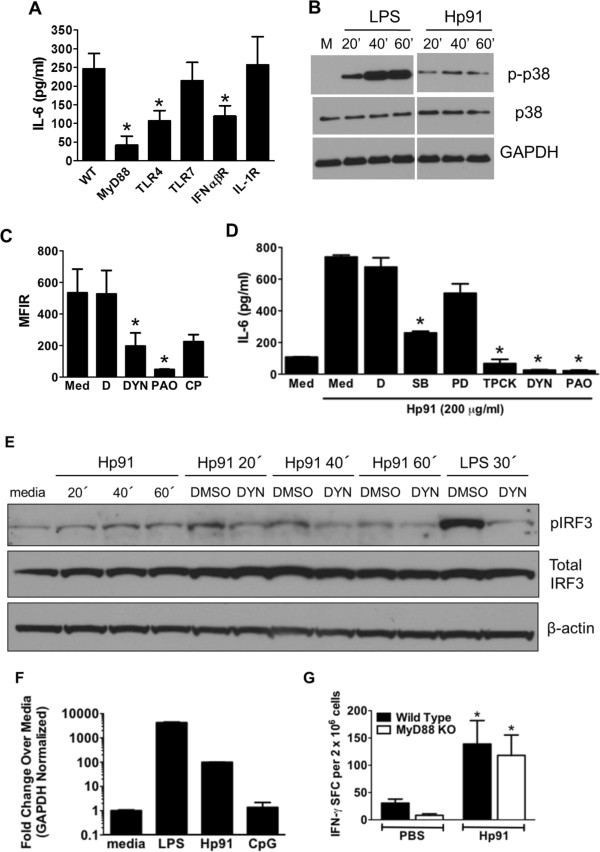
**TLR4, MyD88, and MyD88-dependent and -independent pathways are necessary for Hp91-mediated activation of antigen presenting cells. (A)** BM-DCs from wild type (WT) or knockout mice were exposed to 200 μg/ml Hp91. Supernatants were analyzed for IL-6 by ELISA. Results are mean (±SEM) for N = 3-5. **(B)** J774 macrophages were stimulated for indicated times with 200 μg/ml Hp91, 10 ng/ml LPS or left untreated (M). Lysates were analyzed for p-p38 by WB. Blots were probed with anti-p-p38, anti-p38, and anti-GAPDH antibodies. One of N=4. **(C)** J774 macrophages were pretreated with media (Med), DMSO control (D), Dynasore (DYN) 80 μM, phenylarsine oxide (PAO) 2 μM or chlorpromazine (CP) 100 μM before incubation with 200 μg/ml Cp488-labeled Hp91 for 30 min. Cells were analyzed by flow cytometry. Results are mean (±SEM), N=4. **(D)** J774 macrophages were pretreated with DMSO control (D), SB203580 (SB) 20 μM, PD98059 (PD) 20 μM, TPCK 20 μM, Dynasore (DYN) 80 μM, phenylarsine oxide (PAO) 2 μM, or chlorpromazine (CP) 100 μM then exposed to Hp91. Supernatants were analyzed for IL-6 by ELISA. Data are mean (±SEM), N=3. **(E)** RAW 264.7 macrophages were pretreated with medium, DMSO control, or Dynasore (DYN) 80 μM prior to stimulation for indicated times with Hp91 (200 μg/ml), LPS (10 ng/ml) or media. Cell lysates were analyzed for p-IRF3. Immunoblots were probed with anti-p-IRF3, anti-IRF3, and anti-β-actin antibodies. **(F)** cDNA from stimulated J774 cells were evaluated for IFN-α2 mRNA by qPCR. Values are normalized against endogenous GAPDH controls, in duplicate. **(G)** Wildtype or MyD88-/- knockout mice were immunized with SIINFEKL peptide in PBS with or without Hp91. Splenocytes from immunized mice were cultured with SIINFEKL peptide (2.5 μg/ml). The number of IFN-γ-secreting cells was determined 18 h later. Data are mean (±SEM), 5–10 mice/group. *p < 0.05; Student’s t-test.

Several receptors have been implicated in mediating the responses to HMGB1, including TLR4 [19–22, 24, 26]. Since our data show that receptor-mediated uptake, i.e. clathrin-mediated endocytosis, is clearly involved in the uptake of Hp91 (Figure 2B), we sought to identify the receptor(s) involved in Hp91-induced activation of DCs. Bone marrow (BM)-DCs generated from wild type and various knockout mice were exposed to Hp91. IL-6 secretion was significantly reduced in BM-DCs generated from MyD88-/-, TLR4-/-, and IFNαβR-/- knockout mice (Figure 3A), whereas IL-6 production from BM-DCs generated from TLR7-/- and IL-1R-/- knockout mice was comparable to wild type cells (Figure 3A), indicating that TLR4/MyD88 and IFNαβR are involved in the activation of DC by Hp91.

### Hp91-mediated activation of mouse antigen presenting cells is clathrin- and dynamin-dependent

**Figure 4:**
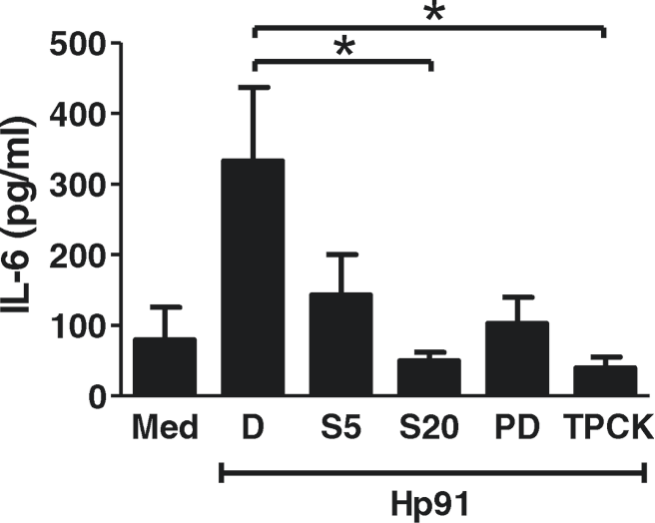
**p38MAPK and NF-κB signaling are necessary for Hp91-mediated IL-6 secretion by dendritic cells.** Immature human DCs were pre-treated for 30 min with DMSO control (D), p38 inhibitor SB203580 at 5 μM (SB5) or 20 μM (SB20), MEK1 inhibitor PD98059 (PD) at 20 μM, or NF-κB inhibitor TPCK at 20 μM prior to exposure to Hp91. Cell culture supernatants from the different groups were collected after 48 h and analyzed for the presence of IL-6 by ELISA. Results are mean (±SEM) for N = 5. *p < 0.05; Student’s t-test.

Since ligand engaged TLR4 is endocytosed in a dynamin-dependent manner for downstream signaling in macrophages exposed to LPS [35], we evaluated if Hp91 signals through a similar TLR4 mechanism as dynamin is part of the machinery for clathrin coated pit endocytosis [36]. The dynamin-dependent endocytosis inhibitor, dynasore, significantly inhibited uptake of Hp91 by macrophages (Figure 3C), which is in agreement with the finding reported for LPS [35]. The clathrin-mediated endocytosis inhibitor phenylarsine oxide also inhibited uptake of Hp91 by macrophages (Figure 3C). In addition, blocking uptake of Hp91 via dynamin or clathrin mediated endocytosis significantly reduced IL-6 secretion (Figure 3D). Since a reduction in Hp91-mediated IL-6 secretion was observed in human DCs following pretreatment with the p38MAPK and NF-κB inhibitors (Figure 4), we evaluated if the same was true for mouse macrophages. As was seen in human DCs, inhibition of p38MAPK or NF-κB signaling cascades significantly lowered the amount of Hp91-stimulated IL-6 secreted from mouse macrophages (Figure 3D), further confirming the involvement of p38MAPK and NF-κB in Hp91 signaling.

### DC activation by Hp91 peptide involves signaling via a p38 MAPK and NF-κB dependent pathway

Our previous findings have shown involvement of the p38MAPK pathway in the HMGB1 B box-induced secretion of IL-6 by human DCs [37]. Pretreatment of human DCs with the p38MAPK inhibitor SB203580, and the NF-κB inhibitor N-p-Tosyl-L-phenylalanine chloromethyl ketone (TPCK), significantly reduced the Hp91-induced IL-6 production in DCs (Figure 4). In contrast, PD98059, a MEK1 inhibitor, failed to significantly decrease the IL-6 production (Figure 4). The p38MAPK pathway was further confirmed by immunoblotting for phosphorylated p38MAPK (p-p38). p-p38 was up-regulated in mouse BM-DCs after 40–60 min of Hp91 stimulation (Figure 3B). In most experiments, up-regulation of p-p38 was observed as early as 20 minutes after Hp91 stimulation. These results suggest that Hp91 induced the DC activation in a p38MAPK and NF-κB pathway dependent manner.

### Hp91 activates IRF3 and subsequent IFNα production in antigen presenting cells

TLR4 can signal through MyD88 dependent and independent pathways, whereas other TLRs are MyD88-dependent. Our data indicate that both pathways might be involved in Hp91 signaling since a decrease in Hp91-induced IL-6 secretion was observed for both MyD88-/- and IFNαβR-/- knockout BM-DCs (Figure 3A). We investigated if Hp91 activated the MyD88 independen signaling pathway by investigating phosphorylation of the downstream interferon regulatory factor 3 (IRF3) transcription factor. The phosphorylation of IRF3 (pIRF3) was up-regulated after Hp91 stimulation as early as 20 min (Figure 3E) and at a maximum around 40 min after stimulation. In addition blocking Hp91 endocytosis with dynasore reduced the phosphorylation of IRF3 at 20, 40, and 60 min (Figure 3E). To further investigate involvement of the MyD88-independent signaling pathway, we evaluated IFN-α mRNA expression in Hp91-stimulated mouse macrophages as this factor should be up-regulated by pIRF3. Although lower than the increase induced by LPS, Hp91 induced a 99-fold increase in IFN-α mRNA levels (Figure 3F). These findings suggest that Hp91 uptake is required for MyD88-independent activation of the IRF3 pathway and production of IFN-α.

### MyD88 signaling is not required for Hp91-elicited CTL responses *in vivo*

Since Hp91 activated both the MyD88 dependent as well as independent pathways (Figure 3A and 3E), we tested if MyD88 was required for Hp91-induced CD8+ CTL responses *in vivo*. We immunized age-matched wild type and MyD88-/- knockout mice with OVA-I (SIINFEKL) peptide co-injected with PBS or Hp91. Both wild type and MyD88-/- knockout mice showed similar antigen-specific cellular immune responses, as demonstrated by increased number of OVA-specific IFN-γ secreting cells (Figure 3G) suggesting that induction of this antigen specific CD8+ CTL response did not require MyD88 signaling. The lack of IL-6 secretion in MyD88-/- DCs after Hp91 exposure indicated that Hp91 can signal through MyD88, whereas the adjuvant effect elicited by Hp91 *in vivo* is MyD88 independent.

## Discussion

Endogenous TLR agonists and inflammatory mediators are attractive candidates as vaccine adjuvants, especially for subunit vaccines that many times are poorly immunogenic, and the mechanism through which these types of adjutants augment immune responses is via the innate immunity. There is a great need for safe and potent adjuvants seeing that and we have previously shown that the 18 amino acid long immunostimulatory peptide Hp91, derived from the B box of the HMGB1 protein, is a potent stimulus for human DCs with the ability to generate a Th1-type immune response *in vitro* [32]. In addition, Hp91 acted as adjuvant *in vivo*, inducing cellular immune responses to peptide and both cellular and humoral immune responses to protein antigen [33]. In this study we characterize the mechanism of action for this adjuvant and show here that Hp91 exerted its immunostimulatory effects on DCs by inducing cellular uptake and activating signaling cascades. The Hp91 peptide was taken up into cells very rapidly via clathrin-mediated endocytosis in a sequence specific manner. Scrambling the amino acid sequence of Hp91 resulted in a great reduction of binding/uptake by cells, indicating that it is neither the total charge nor total hydrophobicity that is important for uptake rather the unique amino acid sequence. Further characterization showed that Hp91 mediated activation of DCs, i.e. IL-6 production *in vitro,* was dependent on TLR4, MyD88, and IFNαβR, whereas MyD88 signaling *in vivo* was not required for activation of CD8+ CTL responses.

Multiple HMGB1 binding and signaling events mediate activation of innate immune responses. The binding and uptake of Hp91 by DCs was a rapid and sequence dependent event and we found that the internalization occurred via clathrin dependent endocytosis. Since clathrin-mediated endocytosis is a receptor dependent uptake, we explored possible receptors involved in the uptake of Hp91. Several receptors have been implicated, including RAGE, TLR4, TLR2, CD24/Siglec-10. HMGB1 has been shown to contribute to LPS-mediated DC maturation via RAGE [18]. TLR2 and TLR4 have been shown to be involved in HMGB1 signaling *in vitro* [19–23]. *In vivo* data have shown binding and signaling through TLR4 to be involved in HMGB1-induced cytokine release, i.e. inflammation leading to tissue damage in the body [20, 38].

The C-terminal motif of HMGB1 (150–183 amino acids) is responsible for RAGE binding [38], whereas the C-terminal end contains the TLR4 binding site. The Hp91 peptide is located in the B box area of HMGB1 protein and contains the TLR4 binding domain and we found that the ability to bind TLR4 was still intact in the Hp91 peptide.

It has been shown that LPS, a TLR4 ligand, binds TLR4, and is subsequently endocytosed together with the receptor [35] and this seems also to be the case for Hp91 peptide. By evaluating IL-6 secretion from knockout mice, we show that Hp91-stimulated activation of DCs is dependent on TLR4 and its downstream adaptor protein, MyD88 and further downstream signaling via p38MAPK and NF-κB. We have previously shown the involvement of the p38MAPK pathway in induction of IL-6 secretion in human DCs by the HMGB1 subunit B box [37] and others have shown that this pathway is involved in HMGB-1 induced activation of neutrophils [39]. This indicates that both the HMGB1 derived peptide and the whole protein have the ability to activate the p38MAPK pathway and that the activating sequence seems to be located within the Hp91 peptide.

Hp91 acts as adjuvant *in vivo*; inducing cellular immune responses to peptide and both cellular and humoral immune responses to protein antigen [33]. Immunization of MyD88-/- mice, using Hp91 as adjuvant, induced cellular immune responses comparable to WT mice. This suggests that even though MyD88 was required *in vitro* for Hp91-mediated IL-6 secretion in DCs, the Hp91-induced cellular immune responses *in vivo* are MyD88-independent. Furthermore, active uptake of Hp91 was required for signaling through this MyD88-independent pathway. Hp91 induces the *in vivo* production of Th1-type cytokines, such as IL-12, and IFN-γ [33]. The Th1 cellular immune response is highly characterized by IFN responses and we found that Hp91- induced a signaling cascade activating the type I IFN pathway via IRF3, leading to elevated expression of IFN-α mRNA. Blocking the type I IFN receptor dampened the immune response to another potential adjuvant, i.e. Poly I:C, indicating that type I IFN play an important role in the immune activation induced by this adjuvant [40] Our data suggest that the activation of DCs by Hp91 is dependent on an autocrine type I IFN feedback loop as cells derived from IFNαβR knockout mouse failed to respond. This combined with the result showing that MyD88 was not necessary *in vivo* for cellular immune responses, suggests that the MyD88-independent pathway plays a prominent role in Hp91 induction of cellular immune responses *in vivo*.

These new findings provide a better understanding of the cellular mechanisms by which the immunogenic peptide induces potent immune responses. This peptide activates myeloid DCs and should be suitable as an adjuvant in cancer immunotherapies as well as vaccines against infectious diseases.

## Electronic supplementary material

Additional file 1: **Uptake of Hp91 is temperature dependent. (A-B)** Immature human DCs were pre-cooled on ice for 30 min, then incubated with biotinylated Hp91 (200 μg/ml) for 30 min at 4, 16, or 37°C. Cells were permeabilized with Cytofix/Cytoperm, stained with Streptavidin-Alexa488, and analyzed by flow cytometry. **(A)** Data are mean (±SEM) of N=3 and **(B)** is a representative result. ***p < 0.001; Student’s t-test. Data are mean (±SEM) of triplicate samples, but data is representative of 3 independent experiments. (PPTX 131 KB)
