# Supplementary material for: TLR4-dependent activation of dendritic cells by an HMGB1-derived peptide adjuvant
Source: J Transl Med. 2014 Aug 14;12:211. doi: 10.1186/1479-5876-12-211 (PMC4261565; doi:10.1186/1479-5876-12-211)
Supplement: Supplementary file 1 — Additional file 1: Uptake of Hp91 is temperature dependent. (A-B) Immature human DCs were pre-cooled on ice for 30 min, then incubated with biotinylated Hp91 (200 μg/ml) for 30 min at 4, 16, or 37°C. Cells were permeabilized with Cytofix/Cytoperm, stained with Streptavidin-Alexa488, and analyzed by flow cytometry. (A) Data are mean (±SEM) of N=3 and (B) is a representative result. ***p < 0.001; Student’s t-test. Data are mean (±SEM) of triplicate samples, but data is representative of 3 independent experiments. (PPTX 131 KB) [file 12967_2014_1920_MOESM1_ESM.pptx]

## Slide 1
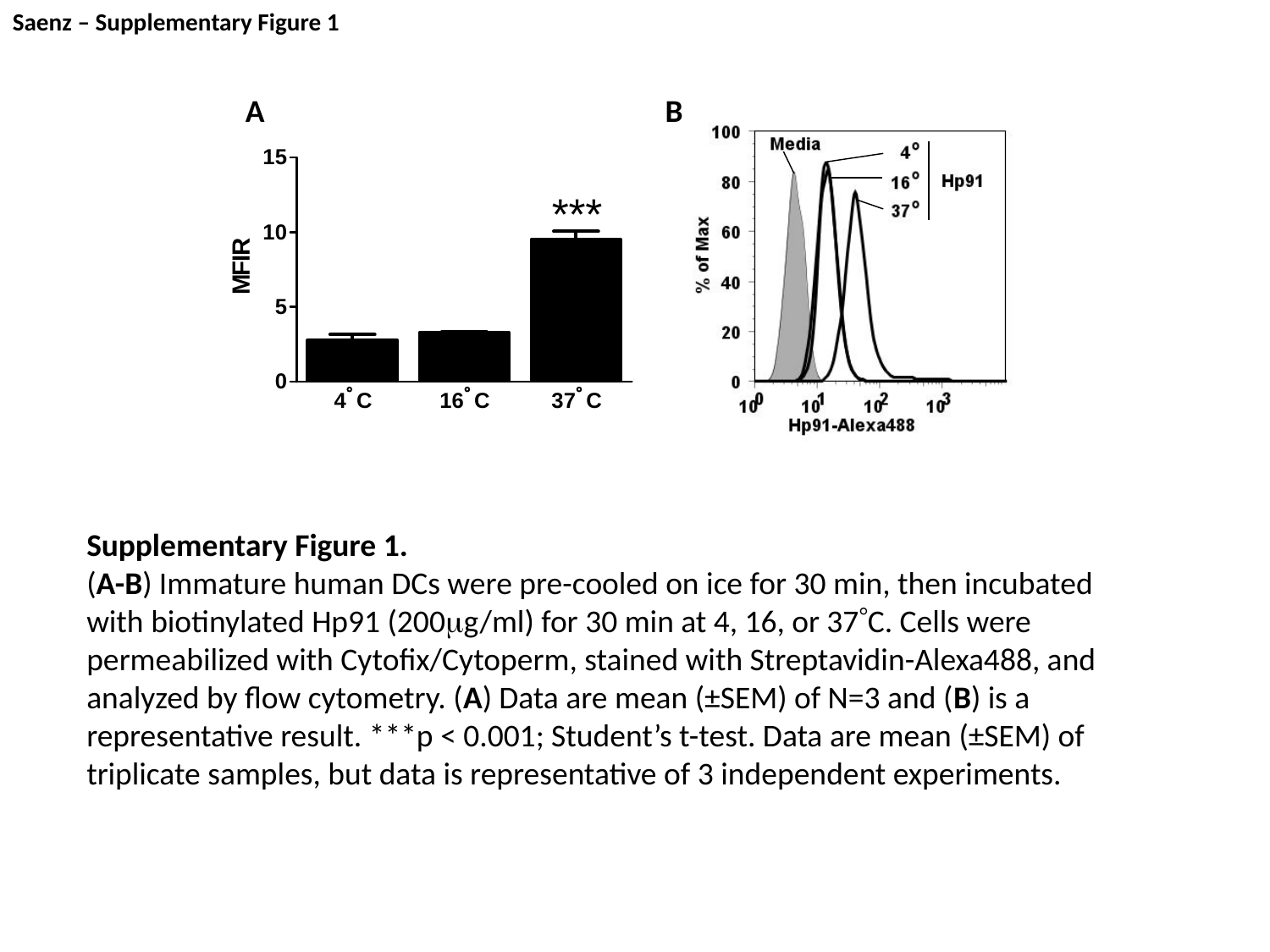

Saenz – Supplementary Figure 1
A
B
Supplementary Figure 1.
(A-B) Immature human DCs were pre-cooled on ice for 30 min, then incubated with biotinylated Hp91 (200μg/ml) for 30 min at 4, 16, or 37°C. Cells were permeabilized with Cytofix/Cytoperm, stained with Streptavidin-Alexa488, and analyzed by flow cytometry. (A) Data are mean (±SEM) of N=3 and (B) is a representative result. ***p < 0.001; Student’s t-test. Data are mean (±SEM) of triplicate samples, but data is representative of 3 independent experiments.
